# Single- and Multi-Locus GWAS Unravels Novel Genomic Regions Related to Low-Phosphate Stress in Cotton Seedlings

**DOI:** 10.3390/plants14121803

**Published:** 2025-06-12

**Authors:** Xianxu Wei, Siyu Yao, Jiangnuo Di, Jiaxin Guan, Aohan Wang, Jie Yang, Luyao Zhang, Yang Liu, Mengyao Liang, Zhihao Niu, Xuan Zhang, Jiarui Xue, Mengxue Shen, Lin Li, Yao Su, Zhengwen Sun

**Affiliations:** 1 State Key Laboratory of North China Crop Improvement and Regulation, Hebei Agricultural University, Baoding 071001, China; 18303016089@163.com (X.W.); y15176739867@sina.com (S.Y.); 13290622175@163.com (J.D.); 15503373512@163.com (J.G.); 15231979806@163.com (A.W.); yangjie0417@hotmail.com (J.Y.); 19833895188@163.com (L.Z.); 13031566901@163.com (Y.L.); mybq06120729@sina.com (M.L.); 13089750423@163.com (Z.N.); 15230273972@139.com (X.Z.); 18330212132@163.com (J.X.); 15931271658@163.com (M.S.); 13931373018@163.com (L.L.); 2 North China Key Laboratory for Germplasm Resources of Education Ministry, Hebei Agricultural University, Baoding 071001, China; 3 Key Laboratory for Crop Germplasm Resources of Hebei Province, Hebei Agricultural University, Baoding 071001, China

**Keywords:** cotton, low phosphorus, GWAS, candidate genes, molecular breeding

## Abstract

Phosphorus (P) is an essential nutrient for plant growth, and low-phosphorus (LP) stress significantly limits cotton productivity. Here, we conducted single- and multi-locus genome-wide association studies (GWASs) on four LP-related traits using 419 upland cotton (*Gossypium hirsutum* L.) accessions genotyped with 2.97 million single-nucleotide polymorphisms (SNPs). Phenotypic analysis reveals substantial variation under LP stress, with LP-SDW showing the highest coefficient of variation (33.69%). The GWASs identified thousands of significant SNPs, including pleiotropic loci associated with multiple traits. Chromosomes A08, D09, and D12 harbored novel associated signals. Multi-locus models significantly enhanced detection sensitivity, identifying 123 SNPs undetected by single-locus approaches. Functional annotations prioritized six candidate genes near associated SNPs, including GhM_A08G1315 (remorin protein) and GhM_D06G1152 (carotenoid cleavage dioxygenase), whose LP-induced expression patterns were validated by qRT-PCR. These genes are implicated in membrane signaling, root architecture modulation, and hormone metabolism. Our findings provide novel genetic insights into LP tolerance and establish a foundation for breeding phosphorus-efficient varieties through marker-assisted selection in cotton.

## 1. Introduction

Cotton is the most important crop of natural fiber in China and the world [1]. Phosphorus, as one of the three major elements for plants, plays an important role in the growth and development of crops [2]. Although soil contains abundant phosphorus, most of it readily combines with metal ions or clay particles to form insoluble phosphates or organic phosphorus, which are unavailable for plant uptake, resulting in low available phosphorus content in the soil and restricting crop growth and development [3]. More than 30% of crops worldwide are affected long-term by the lack of phosphorus in the soil, and now the excessive application of phosphorus fertilizer in agricultural ecosystems leads to rapid accumulation of phosphorus and accelerates its loss to water bodies, causing the eutrophication of water bodies and endangering environmental safety [4]. Upland cotton accounts for over 90% of the total cotton production, and its fiber is the core raw material for the textile industry, which is crucial for the agricultural economy and people’s livelihood. However, upland cotton is highly sensitive to phosphorus deficiency, which severely inhibits its root development, photosynthetic efficiency, and fiber quality, influencing the field and quality, especially as a large amount of phosphate fertilizer is required in poor soil, which exacerbates production costs and environmental pollution risks. Therefore, analyzing the molecular mechanisms of response to low-phosphorus stress at the genomic level is of great significance for cultivating varieties with low-phosphorus tolerance, reducing phosphorus fertilizer application, and developing green agriculture.

Plants adapt to low-phosphorus stress through morphological, physiological, and molecular adjustments. Roots, as the main site for absorbing phosphorus, play an important role in plant adaptation to low-phosphorus stress [5]. Under low-phosphorus stress, plants can regulate the spatial structure of their roots by increasing lateral roots and root hairs, thereby improving phosphorus absorption capacity and efficiency. For instance, wheat can increase the root/shoot ratio by enhancing the metabolism of energy and carbohydrates in roots responding to low-phosphorus stress [6]. In addition, plants can respond to low-phosphorus stress by adjusting physiologically active substances, such as specific secretions, hormones, and assimilates in their roots [7].

Biomass-related traits are quantitative traits that are jointly controlled by multiple genes and the environment in plants [6]. The genetic basis is complex, so it is difficult to deeply explore related genes through traditional breeding. With the rapid development of molecular technology, genome-wide association studies (GWASs) based on the linkage disequilibrium represent powerful tools for dissecting crop agronomic traits [8]. So far, the application of GWASs has successfully accelerated genetic research and discovered genetic variations that control important traits such as fiber quality, yield, disease, and salt resistance in cotton [9]. However, a single-locus GWAS model cannot estimate the joint effect of multiple loci and has relatively low efficacy. Therefore, multi-locus GWAS methods, such as mrMLM, FASTmrEMMA, FASTmrMLM, and IIIVmrMLM, have been developed in recent years [9]. The introduction of multi-locus GWAS methodologies has enhanced the precision of minor-effect locus detection [10], thereby enriching our understanding of the complex genetic basis of low-phosphorus tolerance in cotton [11,12].

In this study, we investigated four related traits (plant height [LP-PH], shoot dry weight [LP-SDW], root dry weight [LP-RDW], and the root-to-shoot ratio [LP-RSR]) under low-phosphorus stress during the seedling stage of 419 upland cotton accessions and performed single- and multi-locus GWAS to identify genomic regions related to these traits based on genotypic data from 2.97 million SNPs. This study provides valuable genetic loci and candidate genes for the molecular design breeding of low-phosphorus tolerance for cotton.

## 2. Results

### 2.1. Phenotypic Analysis of Related Traits Under Low-Phosphate Stress

The traits of 419 upland cotton accessions exhibited widespread phenotypic variation under low and normal phosphorus conditions (Table 1). The continuous distributions of these traits showed typical quantitative characteristics controlled by multiple genes. The variation in the range of these traits was analyzed. The variation ranges of LP-PH, LP-SDW, LP-RDW, and LP-RSR were 2.73~8.52 cm, 0.08~0.60 g, 0.16~0.53 g, and 0.78~4.03, respectively. Among them, LP-SDW had the highest coefficient of variation (CV) in the phenotype, which is 33.69%, and was most affected by low-phosphorus stress. Variation in LP-PH and LP-RDW was small, with 19.65% and 19.75% CVs. For example, Shandaixi, Liao113, and Kuche96515 were P deficiency-tolerant varieties with higher biomass, and CCRI17, Jifeng908, and Baimian1 were P deficiency-sensitive varieties with lower biomass.

**Figure 1:**
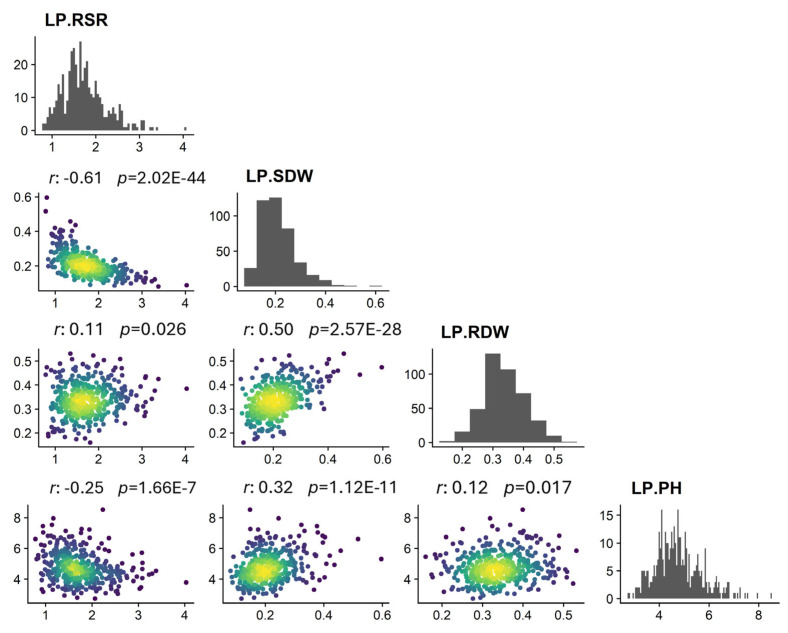
Correlations of four traits under low-phosphorus stress.

We further analyzed correlations among traits (Figure 1). LP-PH correlated positively with the other two traits but negatively with LP-RSR. LP-RDW correlated positively with LP-SDW and LP-RSR. LP-SDW correlated negatively with LP-RSR. These results indicate that cross-links existed among these related traits, which together affected cotton resistance to low-phosphate stress.

### 2.2. Identification of Genetic Loci for Related Traits

**Figure 2:**
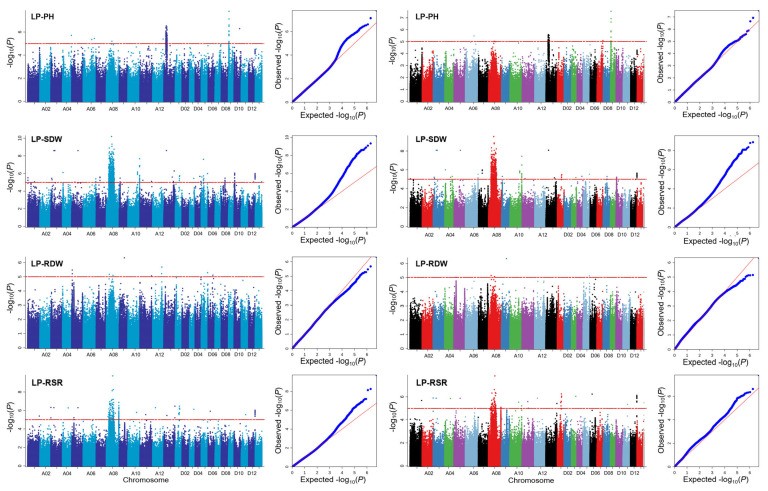
Manhattan and QQ plots of different traits by GWASs. Left side indicates the results from EMMAX, and right side indicates the results from GEMMA.

GWASs were performed to detect significant associated SNPs using single-locus and multi-locus models for four traits under low-phosphorus stress, including LP-PH, LP-SDW, LP-RDW, and LP-RSR (Figure 2). The results show 1491 SNPs significantly associated with these traits based on single-locus models and 125 SNPs based on multi-locus models (Table S1).

**Figure 3:**
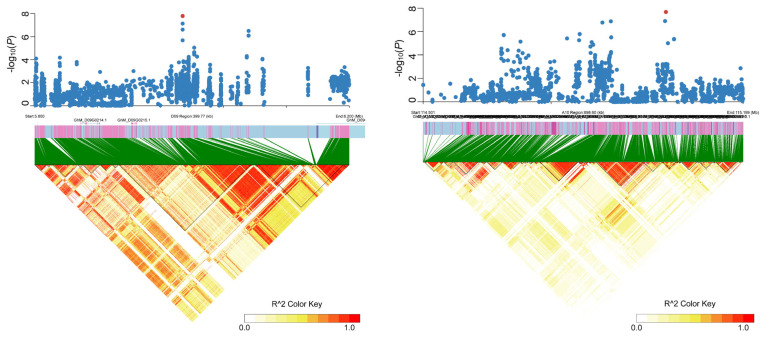
The Local Manhattan plot (**top**) and LD heat map (**bottom**) surrounding the novel associated regions on chromosomes D09 (**left**) and A10 (**right**), respectively. Red points indicate the position of significantly associated SNPs.

For single-locus models, there were 219, 531, 11, and 174 SNPs significantly associated with LP-PH, LP-SDW, LP-RDW, and LP-RSR by EMMAX. There was a total of 531 SNPs significantly associated with LP-SDW, and the highest number of SNPs was distributed on A08. Importantly, a novel significantly associated signal was found in the A10 chromosome. In addition, a significantly associated signal with LP-PH was found in D09 chromosomes with the highest number (13) of SNPs (Figure 3). Furthermore, 38, 436, 6, and 76 SNPs were significantly associated with the four traits by GEMMA. LP-SDW also had the highest number of significantly associated SNPs.

A total of 531 SNPs were simultaneously detected by EMMAX and GEMMA. There were eight SNPs on D09 significantly associated with LP-PH. LP-RSR was detected simultaneously in A08, D01, D03, and D12. There was a total of 12 SNPs associated with LP-RSR and LP-SDW in D12. We further analyzed the SNPs associated with four traits. A total of 62 and 15 SNPs were significantly correlated with these traits under low-phosphorus stress. For the multi-locus model, we identified 123 SNPs undetected by single-locus approaches, including the two SNPs on A08 and D12 simultaneously associated with LP-RSR and LP-SDW.

### 2.3. Candidate Gene Prediction

A total of 3152 genes were screened on the flank of significantly associated SNPs (Table S1). Through functional annotation of these genes and related research reports, we further identified six genes involved in environmental stress response or regulation as potential candidate genes for response to low-phosphorus stress.

A novel signal having five SNPs associated with LP-PH was found in D09. We identified a candidate gene in this region. The gene GhM_D09G0217 is an uncharacterized protein without relative reports. In A08, eight SNPs were detected to be significantly associated with LP-SDW and were located near the gene GhM_A08G1315. The gene was annotated as a remorin protein. Remorin proteins are a plant-specific oligomeric filamentous protein family that is linked to the plasma membrane/lipid raft and is located in the membrane microdomain, playing a crucial role in plant growth and development, signal transduction, and stress response [13].

In A10, there were two candidate genes associated with both LP-SDW and LP-RSR. GhM_A10G2833 and GhM_A10G2845 encode mannose-1-phosphate guanylyltransferase (*MPG*) and mitochondrial substrate carrier family protein B (*McfB*), respectively. *MPG* is one of the key enzymes in plant and microbial metabolic pathways involved in various metabolic reactions, including starch synthesis, polysaccharide synthesis, and the metabolism and regulation of other biomolecules [14]. *McfB* plays an important role in mitochondrial metabolism and is capable of transporting various substrates that play critical roles in energy production and cellular metabolism [15].

GhM_D06G1152 is associated with LP-RSR. The gene encodes carotenoid cleavage dioxygenase (*CDD*). *CDD* is involved in the metabolism of carotenoids in plants and animals, and strigolactones (SLs) are a class of plant hormones derived from carotenoids that can regulate plant development [16]. In D12, we identified 12 SNPs simultaneously associated with LP-SDW and LP-RSR. The candidate gene GhM_D12G3169 encodes guanine nucleotide-binding protein subunit beta-like protein (*GNB1L*). *GNB1L* participates in cellular signal transduction by binding to G protein-coupled receptors. It can regulate various intracellular signaling pathways, thereby affecting the physiological and pathological processes of cells [17].

### 2.4. Validation of Candidate Gene Expression

**Figure 4:**
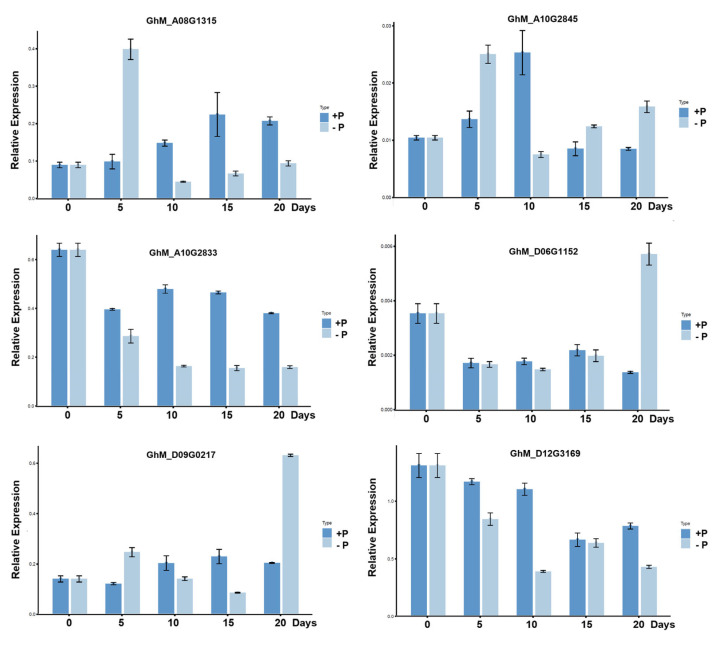
Relative expression patterns by qPCR between low-phosphorus stress and normal phosphorus in roots. Values are means with standard deviation (SD) (*n* = three biological replicates). +P: 1.0 mmol/L, −P: 0 mmol/L.

To validate candidate genes related to low-phosphorus stress, the expression levels of six candidate genes were analyzed. The results show that there were significant differences in these genes at different times of root development between low-phosphorus stress and normal phosphorus (Figure 4). After low-phosphorus treatment, the qRT-PCR results show that the expression levels of GhM_D06G1152 and GhM_D09G0217 in roots under LP stress were significantly upregulated at 20 d compared to normal phosphorus conditions. The expression level of GhM_A08G1315 and GhM_A10G2845 was significantly higher at 5 d under low phosphorus. In contrast, the expression of GhM_A10G2833 and GhM_D12G3169 gradually decreased with time under low phosphorus.

The candidate genes identified that may be involved in response to low-phosphorus stress provide valuable references for further elucidating the molecular mechanisms of tolerance to low-phosphorus stress in cotton. However, the function of the candidate genes still needs to be further validated.

## 3. Discussion

In this study, we used phenotypes of four related traits of 419 cotton accessions under low phosphorus and conducted GWASs to identify associated SNPs and genes using single-locus and multi-locus models, providing useful information for revealing the mechanisms of tolerance to low phosphorus and molecular marker-assisted breeding in cotton.

The seedling stage is a critical period for phosphorus absorption in cotton growth [5]. In this study, the phenotypic variation of these traits during the seedling stage was used to reflect phosphorus utilization efficiency and relative ability to tolerate low phosphorus. The GWASs’ results show that 1491 SNPs distributed throughout the genome were significantly associated with traits under low-phosphorus stress, with a total of 531 SNPs simultaneously detected by two single-locus models. Many candidate genes were identified through these SNP loci, of which six genes were validated. These genes related to the growth and development of crops under environmental stress have not been reported in cotton [18], which can provide a reference for studying the genetic basis of response to low-phosphorus stress.

The growth process of cotton is regulated by plant hormones and influenced by environmental factors, among which the root morphology of plants under low-phosphorus stress is closely related to gibberellin [19]. The SNP marker D06:21262217 (C > T) was associated with LP-RSR and located in GhM_D06G1152 encoding *CDD*. Strigolactone was discovered in the 1960s and is now known as the seventh type of plant hormone. It is derived from carotenoids secreted by plant roots through enzymatic reactions and can inhibit lateral bud growth. Knocking out the gene *ZmCCD8* encoding carotenoid cleavage dioxygenase in maize affected the biosynthesis of SL, resulting in thinner stems, shorter internodes, delayed root development, and a reduction of about 10% in plant height [20]. In addition, the expression of *CCD* genes is also regulated by plant hormones such as auxin and gibberellin, which further affect the growth and development process of plants [21].

The genetic research on plant response to stress mainly focuses on signal transduction and gene expression regulation at the molecular level [22,23,24]. Stress response genes can be classified into two categories based on the function of their encoded products: one is genes encoding functional proteins that directly or indirectly protect plant cells, and the other is genes related to signal transduction and transcriptional regulation [25]. This study found that GhM_A08G1315 encoding *remorin* was detected in LP-SDW. Remorin proteins are a newly discovered plant-specific protein family associated with the plasma membrane/lipid rafts. They can be phosphorylated in response to the activation of plant defense and growth development genes and may participate in various signaling pathways in plants, such as salt stress, drought stress, and disease-resistance signaling pathways, particularly in their interaction with intercellular filaments, involvement in signal transduction, and research on disease resistance [13]. For example, studies have found that remorin protein mediates the closure of intercellular filaments by regulating lipid distribution, thereby affecting plant defense responses against pathogens [26].

This study found that the upregulation of GhM_A08G1315 (*remorin*) under LP stress suggests its potential role in enhancing membrane signaling efficiency, a mechanism conserved across plant species [13], indicating it may have a positive regulatory effect on the low-phosphorus tolerance of cotton seedlings and may be used as a candidate gene for further functional validation.

## 4. Materials and Methods

### 4.1. Materials and Experimental Processing

The 419 upland cotton accessions were collected and preserved by the Cotton Genetics and Breeding Research Laboratory of Hebei Agricultural University. Planted under a dry shed at the experimental base of Hebei Agricultural University, this experiment adopted the potted planting method, with multiple pieces of gauze placed at the bottom of the pot to prevent root growth. Vermiculite was used as the culture medium, with four seeds per pot, and all samples were tested using a completely randomized block design. After the cotyledons had fully expanded (about 10 days), two seedlings with consistent growth in each pot were selected, and the others were removed. Nutrient solutions were prepared using a modified Hoagland formulation, with 1.0 mM KH_2_PO_4_ for the control and 1.0 mM KCl replaced KH_2_PO_4_ for LP treatment. All other components were the same. The pH was adjusted with NaOH/HCl to 6.0 ± 0.1. The experiment was replicated twice, with three pots of seedlings per replicate.

### 4.2. Phenotypic Identification

After 30 days of low-phosphorus treatment, phenotyping was carried out. Plant height (PH, cm) was measured from the cotyledonary node to the top of the main stem. The seedlings were separated from the cotyledons, and the aboveground and root parts were placed in a 105 °C oven for 30 min of withering. Then, they were dried at 65 °C to constant weight, and the root dry weight (RDW, g) and aboveground dry weight (SDW, g) were measured, and the root-to-shoot ratio (RSR, RDW/SDW) was calculated. Statistical analysis of all data was performed using SPSS Statistics 19.0 software and plotted using GraphPad Prism 9.

### 4.3. GWASs and Candidate Gene Identification

GWASs were performed to identify genomic variants using the data described by our previous study [27]. A total of 2,970,970 population SNPs (MAF ≥ 0.05; missing rate ≤ 0.2) obtained from our previous resequencing cotton accessions (average 10.65-fold) were used in GWAS analysis using the single-locus model GEMMA (0.98.5) and EMMAX (ver. 20120210) software and the multi-locus model IIIVmrMLM package, with −log_10_ (*P*) > 5.00, to determine the association between SNPs and traits. Genes flanking 325 kb of the significantly associated SNP were screened as candidates based on the reported upland cotton NDM8 reference genome [27]. Manhattan and QQ plots were drawn using R 4.3.2 software.

### 4.4. Candidate Gene Expression Analysis

Expression analysis of candidate genes was conducted under low- and normal phosphorus treatments [28]. Firstly, the seeds were placed into a germination box filled with quartz sand. When the cotyledons were fully expanded, they were transferred into a 1/2 concentration Hoagland nutrient solution. After 3 days, the nutrient solution was replaced by a low-phosphorus nutrient solution (+P: 1.0 mmol/L, −P: 0 mmol/L). The roots were sampled at 0 d (day), 5 d, 10 d, 15 d, and 20 d and immediately frozen in liquid nitrogen and stored at −80 °C for RNA extraction with three replicates after treatment. Total RNA was extracted using the FastPure Plant Total RNA Isolation Kit (Vazyme, Nanjing, China), and cDNA was obtained using the Hifair^®^ III 1st Strand cDNA Synthesis SuperMix (Yeasen, Shanghai, China). The qRT PCR reaction system and procedure were conducted using the Hieff^®^ qPCR SYBR Green Master Mix (Yeasen, Shanghai, China), and the reaction was performed on a Roche LightCycler 96. *GhUBQ14* was used as an internal reference to calculate the relative gene expression level, with three biological replicates in each, using the formula 2^−ΔΔCT^ method [29]. The primers are listed in Table S2.

## 5. Conclusions

This study integrated single- and multi-locus GWASs to dissect the genetic basis of LP tolerance in cotton, identifying thousands of significant SNPs and prioritizing many candidate genes. Expression analysis revealed LP-induced dynamics of these genes, implicating roles in root architecture, hormone signaling, and membrane microdomain regulation. The upregulation of *remorin* (GhM_A08G1315) and carotenoid cleavage dioxygenase (GhM_D06G1152) highlighted conserved stress adaptation mechanisms. These findings provide critical genetic targets for breeding phosphorus-efficient cotton varieties and promoting sustainable agriculture.

## Figures and Tables

**Table 1:** Phenotypic variation of four traits under low and normal phosphorus conditions.

Trait	Max.	Min.	Mean	SD	CV	*H* ^2^_b_
LP-PH (cm)	8.52	2.73	4.75	0.93	19.65	0.90
NP-PH (cm)	13.84	3.54	7.57	1.65	21.80	
LP-SDW (g)	0.60	0.08	0.21	0.07	33.69	0.90
NP-SDW (g)	0.68	0.18	0.43	0.09	20.71	
LP-RDW (g)	0.53	0.16	0.34	0.07	19.75	0.76
NP-RDW(g)	0.77	0.22	0.44	0.09	19.86	
LP-RSR	4.03	0.78	1.73	0.50	28.76	0.75
NP-RSR	1.99	0.52	1.08	0.23	21.63	

LP/NP-PH, LP/NP-SDW, LP/NP-RDW, and LP/NP-RSR indicate plant height, shoot dry weight, root dry weight, and root–shoot ratio under low/normal phosphorus conditions. SD: standard deviation; CV: coefficient of variation; *H*^2^_b_: broad-sense heritability.

## Data Availability

The authors confirm that the data supporting the findings of this study are available within the article and its Supplementary Materials.

## Supplementary Materials

The following supporting information can be downloaded at: https://www.mdpi.com/article/10.3390/plants14121803/s1, Table S1: The significant associated SNPs identified by single-locus and multi-locus models; Table S2: The list of the primers used in the study.

## References

[1] Smith A., Brown T. (2020). Global Cotton Production and Trade.

[2] Marschner H. (2012). Mineral Nutrition of Higher Plants.

[3] Chiou T.J., Lin S.I. (2011). Signaling network in sensing phosphate availability in plants. Annu. Rev. Plant Biol..

[4] Alewell C., Ringeval B., Ballabio C., Robinson D.A., Panagos P., Borrelli P. (2020). Global phosphorus shortage will be aggravated by soil erosion. Nat. Commun..

[5] Péret B., Desnos T., Jost R., Kanno S., Berkowitz O., Nussaume L. (2014). Root architecture responses: In search of phosphate. Plant Physiol..

[6] Wang J., Qin Q., Pan J., Sun L., Sun Y., Xue Y., Song K. (2019). Transcriptome analysis in roots and leaves of wheat seedlings in response to low-phosphorus stress. Sci. Rep..

[7] López-Arredondo D.L., Leyva-González M.A., González-Morales S.I., López-Bucio J., Herrera-Estrella L. (2014). Phosphate nutrition: Improving low-phosphate tolerance in crops. Annu. Rev. Plant Biol..

[8] Liu X., Huang M., Fan B., Buckler E.S., Zhang Z. (2016). Iterative usage of fixed and random effect models for powerful and efficient genome-wide association studies. PLoS Genet..

[9] Yasir M., Kanwal H.H., Hussain Q., Riaz M.W., Sajjad M., Rong J., Jiang Y. (2022). Status and prospects of genome-wide association studies in cotton. Front. Plant Sci..

[10] Xu Y., Yang T., Zhou Y., Yin S., Li P., Liu J., Xu S., Yang Z., Xu C. (2018). Genome-wide association mapping of starch pasting properties in maize using single-locus and multi-locus models. Front. Plant Sci..

[11] Han Z., Ke H., Li X., Peng R., Zhai D., Xu Y., Wu L., Wang W., Cui Y. (2023). Detection of epistasis interaction loci for fiber quality-related trait via 3VmrMLM in upland cotton. Front. Plant Sci..

[12] Shen J., Yuan L., Zhang J., Li H., Bai Z., Chen X., Zhang W., Zhang F. (2011). Phosphorus dynamics: From soil to plant. Plant Physiol..

[13] Marín M., Ott T. (2012). Phosphorylation of intrinsically disordered regions in remorin proteins. Front. Plant Sci..

[14] Lukowitz W., Nickle T.C., Meinke D.W., Last R.L., Conklin P.L., Somerville C.R. (2001). *Arabidopsis cyt1* mutants are deficient in a mannose-1-phosphate guanylyltransferase and point to a requirement of N-linked glycosylation for cellulose biosynthesis. Proc. Natl. Acad. Sci. USA.

[15] Kretzschmar T., Kohlen W., Sasse J., Borghi L., Schlegel M., Bachelier J.B., Reinhardt D., Bours R., Bouwmeester H.J., Martinoia E. (2012). A petunia ABC protein controls strigolactone-dependent symbiotic signalling and branching. Nature.

[16] Yoo H.J., Chung M.Y., Lee H.A., Lee S.B., Grandillo S., Giovannoni J.J., Lee J.M. (2023). Natural overexpression of CAROTENOID CLEAVAGE DIOXYGENASE 4 in tomato alters carotenoid flux. Plant Physiol..

[17] Syrovatkina V., Alegre K.O., Dey R., Huang X.Y. (2016). Regulation, Signaling, and Physiological Functions of G-Proteins. J. Mol. Biol..

[18] Iqbal A., Dong Q., Wang X., Gui H., Zhang H., Zhang X., Song M. (2023). Phosphorus and carbohydrate metabolism contributes to low phosphorus tolerance in cotton. BMC Plant Biol..

[19] Iqbal B., Kong F., Ullah I., Ali S., Li H., Wang J., Khattak W.A., Zhou Z. (2020). Phosphorus application improves the cotton yield by enhancing reproductive organ biomass and nutrient accumulation in two cotton cultivars with different phosphorus sensitivity. Agronomy.

[20] Yang Q., Li Z., Li W., Ku L., Wang C., Ye J., Li K., Yang N., Li Y., Zhong T. (2013). CACTA-like transposable element in ZmCCT attenuated photoperiod sensitivity and accelerated the postdomestication spread of maize. Proc. Natl. Acad. Sci. USA.

[21] Hou X., Rivers J., León P., McQuinn R.P., Pogson B.J. (2016). Synthesis and function of apocarotenoid signals in plants. Trends Plant Sci..

[22] Vance C.P., Uhde-Stone C., Allan D.L. (2003). Phosphorus acquisition and use: Critical adaptations by plants for securing a nonrenewable resource. New Phytol..

[23] Rouached H., Arpat A.B., Poirier Y. (2010). Regulation of phosphate starvation responses in plants: Signaling players and cross-talks. Mol. Plant.

[24] Nacry P., Canivenc G., Muller B., Azmi A., Van Onckelen H., Rossignol M., Doumas P. (2005). A role for auxin redistribution in the responses of the root system architecture to phosphate starvation in Arabidopsis. Plant Physiol..

[25] Zhang H., Zhu J., Gong Z., Zhu J.K. (2022). Abiotic stress responses in plants. Nat. Rev. Genet..

[26] Jarsch I.K., Ott T. (2011). Perspectives on remorin proteins, membrane rafts, and their role during plant-microbe interactions. Mol. Plant Microbe Interact..

[27] Ma Z., Zhang Y., Wu L., Zhang G., Sun Z., Li Z., Jiang Y., Ke H., Chen B., Liu Z. (2021). High-quality genome assembly and resequencing of modern cotton cultivars provide resources for crop improvement. Nat. Genet..

[28] Luo B., Zhang G., Yu T., Zhang C., Yang G., Luo X., Zhang S., Guo J., Zhang H., Zheng H. (2024). Genome-wide association studies dissect low-phosphorus stress response genes underlying seedling traits in maize. Theor. Appl. Genet..

[29] Vandesompele J., De Preter K., Pattyn F., Poppe B., Van Roy N., De Paepe A., Speleman F. (2002). Accurate normalization of real-time quantitative RT-PCR data by geometric averaging of multiple internal control genes. Genome Biol..

